# Development of a family caregiver needs-assessment scale for end-of-life care for senility at home (FADE)

**DOI:** 10.1371/journal.pone.0222235

**Published:** 2019-09-11

**Authors:** Midori Saito, Etsuko Tadaka, Azusa Arimoto

**Affiliations:** 1 Kanagawa Ward Medical Association Visiting Nursing Station, Yokohama, Kanagawa, Japan; 2 Department of Community Health Nursing, Graduate School of Medicine, Yokohama City University, Yokohama, Kanagawa, Japan; Universitat d'Alacante, SPAIN

## Abstract

**Aim:**

This study aimed to develop a “family caregiver needs-assessment scale for end-of-life care for senility at home” (FADE) and examine its reliability and validity.

**Method:**

A draft item pool was developed based on a literature review, and simplified to 30 items in four domains. Next, the item pool was reviewed by four visiting nurses and four researchers and refined to 15 items. A cross-sectional study was then conducted using a self-reported questionnaire. Questionnaires were sent to 2703 visiting nurses. The survey questions included participants’ basic demographic information, the importance of each item according to a modified scale, basic demographics for cases of death by senility at home, satisfaction with each item of the modified scale in an example case, and assessment of the case using the Japanese version of the Support Team Assessment Schedule (STAS-J). Internal consistency was assessed using Cronbach’s alpha. Construct validity was confirmed using confirmatory factor analysis, and correlation between the new scale and the STAS-J was used to assess criterion-related validity.

**Results:**

In total, 461 visiting nurses provided valid responses. The exploratory and confirmatory factor analyses identified 12 items from two factors: “Needs for adaptation to senility bereavement” and “Needs for essential skills in supporting a dignified death by senility.” The final model showed appropriate index values: standardized root mean residual = 0.057, Tucker–Lewis index = 0.920, Akaike information criterion = 191.6, and Bayesian information criterion = 298.2. Cronbach’s alpha for the entire scale was 0.908, and was above 0.840 for each factor. The correlation coefficient between STAS-J and the entire scale was 0.259–0.427 (p<0.001).

**Conclusions:**

The FADE scale showed acceptable internal consistency and concurrent validity. The scale can help clarify issues and desires that present themselves at home related to adaptation to senility bereavement and essential skills in supporting a dignified death by senility. Addressing these issues and desires is expected to reduce caregivers’ anxiety and burden, and means the older adults under their care may be respected and enabled to live with dignity and peace.

## Introduction

Societal aging is a phenomenon common to nearly all developed countries. In the 100 years between 1950 and 2050, the proportion of the populace made up by older adults is predicted to increase by 13 to 15 percentage points in countries including France, Sweden, and the United States [1]. Population aging is projected to have a profound effect on societies, underscoring the fiscal and political pressures that the health care, old-age pension, and social protection systems of many countries are likely to face in coming decades; therefore, it is necessary to decrease public health expenses [2]. In all European Union countries, the demand for informal care is high and will further increase because of the aging population, as an aging society results in heavy pressures on families and on welfare state regimes [3]. In Europe, informal caregiving provided to people aged 50 years or older by a younger relative accounts for three-quarters of all long-term care [4]. Most patients prefer to die at home [5], so it is essential to prepare caregivers to provide dignified end-of-life care at home [6]. Therefore, it is beneficial for all industrialized countries to understand how to satisfy caregiver needs.

In Japan, the proportion of the populace made up by older adults rose from 12.0% in 1990 to 23.0% in 2010, and is estimated to rise to 33.4% by 2035 and 39.9% by 2060 [1]. The primary cause of death associated with this drastic increase over such a short time is predicted to be death from senility.

Death by senility is defined in Chapter 21 of the International Classification of Diseases, 11th revision [7] as death due to “symptoms, signs or clinical findings, not elsewhere classified.” The Japanese Ministry of Health, Labour and Welfare defines it as “Death of an older adult individual unattributable to other causes; also called natural death.” Death by senility was the fifth most common cause of death in Japan in 2015, but became the fourth most common in 2017 and the third most common in 2018 [8]. Therefore, the order of death from senility is rising year by year. In the near future, in countries like Japan with a long life-expectancy, the primary cause of death will be senility; in Japan 101,396 people died from senility in 2017 [8].

With the predicted increase in death from senility in Japan, it is estimated that there will be 400,000 deaths occurring annually in hospitals from around 2025 [9], which is an unacceptably high number. To adapt to this problem, the Ministry of Health, Labour and Welfare has engaged in securing and maintaining high-quality standards of care for at-home and visiting nursing, thereby effectuating the “promotion of care that is in line with the wishes of the citizenry” [10]. However, the rate of home-deaths in Japan in 2017 remained at 13.2% [11], despite the majority of Japanese people desiring end-of-life care for senility at home [12]. The reason for this critical gap may be the burden on the caregiving family required to provide end-of-life care at home for patients with senility. Previous studies have noted that the burden of family care is the most important factor related to death at home, and therefore it is necessary to develop home care nursing specifically to meet the needs of family caregivers and their burden in providing end-of-life care for senile patients at home [13], as well as meeting the needs and desires of individual older adults in their last days. Providing these needs and desires with dignity at home is not a rare personal problem, but a large social problem in a death-ridden society.

Lynn [14] categorized the diseases that emerge at the end of a person’s life into three broad types: cancer, organ failure, and senility/dementia. While the average care periods for cancer and organ failure are 2 months and up to 6 months, respectively, the average care period for senility and dementia is far longer [14]. As the number of older adults with progressing senility increases, the care periods required from caregivers will lengthen. Increases in caregiver burdens, whether physical, psychological, financial, or societal, are expected to accompany this lengthening [15].

To fulfill the wishes of many older people and their families (i.e., the desire to die of old age at home), it is vital that a means to lessen these burdens on a caregiving family is considered. Previous research revealed that the physical, societal, psychological, and spiritual needs of terminal patients and their caregivers change according to the progress of their conditions [16]. Consequently, it is necessary for medical professionals to grasp the progress of their patients’ conditions and respond to their multidimensional needs accordingly [16]. Similarly, medical professionals also have to grasp and respond to the multidimensional needs of the family-caregivers of patients with progressing senility [17]. It has also been noted that eliciting any concerns early and focusing on the family-caregivers’ needs are likely to lead to better outcomes post bereavement [18]. However, a scale for evaluating these needs does not currently exist. Although various concepts and scales exist for family members of terminal patients with any sort of illness [5], for families of patients with cerebrovascular illness [19], and for families of patients with dementia [20], there is no published concept and scale for family members of senile patients. Moreover, scales evaluating caregiver burden [21], caregiver grief [22], and caregiver quality of life [23] also exist, but these scales are generalized to caregivers of any sort of older adult individual. Therefore, there is no family caregiver need-assessment scale for end-of-life care of senile patients at home.

The objective of this research was therefore to develop a tool for visiting nursing professionals to use when assessing the needs of a family caregiver providing end-of-life care for a senile patient at home, the “Family caregiver needs-assessment scale for end-of-life care for senility at home” (FADE), and to examine its reliability and validity. Visiting nurses were targeted because objective needs assessment by a medical professional is necessary for end-of-life care at home. The visiting nurse is the medical professional with the most opportunity to contact older adults receiving end-of-life care and their family caregivers.

## Materials and methods

### Phase 1: Developing the scale

First, the researchers developed a pool of items based on a literature review. From the perspective of the needs of family caregivers of older adults, PubMed (1946–2018) and Ichushi-Web (1970–2018) were searched for articles about the process of senility and the needs of family-caregivers using specific keywords: senility, end-of-life, older adults, needs, caregiver, family, non-cancer, and scale. This identified 26 articles, of which three had an emphasis on the theme of family caregiver needs [5][19][20]. The item inclusion criteria were based on four viewpoints: 1) care as senility progresses, 2) social resources, 3) family function, 4) acceptance of death and coping at the time of death. Using these four viewpoints as domains, the draft item pool was reviewed and modified several times by the researchers and refined to include 30 items.

Next, the pool of items was reviewed by four visiting nurses and four researchers to assess content validity, face validity, and its practical usefulness in a home care setting via interviews or questionnaires. The visiting nurses included those certified in visiting nursing and an expert administrator of a home-visit nursing care station with more than 20 years’ experience. The researchers were professors or the home care nursing foundation’s researcher from the department of home care nursing, gerontological nursing, or community health nursing, and were also those who developed the measurements [24]. The wording of each item was revised according to reviewers’ recommendations. Consequently, we initially excluded items assessed as “not important” by more than one expert. Thus, the modified scale was refined to 15 items, and one domain (“social resources”) was excluded because it was not specific to senility.

### Phase 2: Validating the scale

#### Study participants

The principle survey involved 2,703 visiting nurses who had experienced giving support to family caregivers of older adults who had died of senility at home. Each nurse worked at a home-visit nursing care station in one of three prefectures in Japan (one participant per facility). The self-administered questionnaire was posted to the participants. Data were collected in three prefectures (Osaka, Tokyo, Kanagawa) because these were the top three prefectures for population and the number of home-visit nursing care stations. The institutions were selected from publicly available information lists. The researchers sent informed consent letters and questionnaires to administrators and eligible participants at each institution. Each participant was asked to complete the voluntary self-administered anonymous questionnaire between September and October 2018. Of the participants contacted, 536 (19.8%) responded, with 461 (17.1%) providing questionnaires with valid responses suitable for the analysis.

#### Measures

Participants’ demographic characteristics included gender, age, years of work experience as a nurse, years of work experience as a visiting nurse, and the number of cases of support for family caregivers of older adult individuals who died of senility at home.

The importance of each FADE scale item was rated on a 4-point Likert-type scale: “Not important = 1,” “Not important to a certain extent = 2,” “Important to a certain extent = 3,” and “Important = 4.” There was also an “I don’t know” option, which was given a rating of 0, to identify the items that professionals felt were not important or did not make sense.

Participants were asked to recall one example of a case of supporting the family caregiver of an older adult who died of senility at home, and assessed the goodness of fit of each item for the case on a 4-point Likert-type scale: “Satisfied = 0,” “Satisfied to a certain extent = 1,” “Unsatisfied to a certain extent = 2,” and “Unsatisfied = 3.”

The older adults’ demographic characteristics included gender, age at the time of death, and support needed at the time of death of that individual. The caregivers’ characteristics were gender, age, relationship, and care period.

To assess the concurrent validity of the scale, the participants were also asked to assess each item in the “Japanese version of the Support Team Assessment Schedule” (STAS-J) [25]. The STAS is a rating scale for hospice and palliative care developed by Higginson et al. in the UK [26]. The Japanese version was developed by Miyashita et al. in 2004 [25], and revised to its third edition in 2007 [27]. This scale consists of nine items: pain control, control of symptoms other than pain, patient’s anxiety, family anxiety, patient’s medical condition recognition, family medical condition recognition, communication between patients and their families, communication among professionals, and medical staff communication to patients and families. Each item is composed of five stages from 0 (No problem) to 4 (Most severe); respondents see text explaining each stage and select the closest one. Each item is interpreted individually, and the summing of items to give a total score is not recommended. The weighted κ statistic for the inter-rater reliability of each item ranges from 0.53 to 0.77, and the κ statistic for the intra-rater reliability of each item ranges from 0.64 to 0.85 [25].

In this study, the items assessed were: family anxiety (item 4), family medical condition recognition (item 6), communication between patients and their families (item 7), and medical staff communication to patients and families (item 9) [27]. These items were selected because they mean that support is needed for family caregivers to fulfill caregivers’ needs.

#### Ethical considerations

This study was conducted with the approval of the Institutional Review Board of the Medical Department of Yokohama City University School (Approval No. A180700008).

#### Statistical analysis

All analyses were conducted using IBM SPSS Statistics 25.0 and Amos 24.0.

Item and exploratory factor analyses were conducted to investigate the reliability and convergent validity of the scale. The criteria for item analysis included pass efficiency (average score ≤2.0 points), rates of response difficulty (unknown and non-respondents ≥5%), distribution (“Important to a certain extent” and “Important” <85% of the sample), good–poor analysis (no significant differences between the highest- and lowest-scoring groups), item-total analysis (correlation coefficient <0.3), and correlations of each item (correlation coefficient >0.7).

The total sample (n = 461) was randomly divided into two split samples for cross-validation: group 1 (n = 231) for performing exploratory factor analysis and group 2 (n = 230) for performing confirmatory factor analysis. The items remaining after item analysis were examined using exploratory factor analysis (main factor analysis) with promax rotation. With reference to eigenvalues and scree plots, it was estimated that there were two or three factors. The exploratory factor analysis was then repeated, presuming two or three factors and excluding items with item loadings ≤0.41. When the differences among the factor loadings became clear, it was possible to interpret the factors theoretically. Factor reliability was determined according to a Cronbach’s alpha ≥0.70. Confirmatory factor analysis was then conducted to verify the construct validity. The standardized root mean residual (SRMR), Tucker–Lewis index (TLI), Akaike information criterion (AIC), and Bayesian information criterion (BIC) were used to evaluate the data–model fit. Values of SRMR ≤0.08 and TLI ≥0.95 were considered to indicate acceptable model fit [28]. Furthermore, criteria-related validity was examined using the STAS-J.

## Results

### Respondent characteristics

Table 1 shows the characteristics of the respondents. A total of 92.8% were female. The median age was 49.0 years, average work experience as a nurse was 23.7 years, average work experience as a visiting nurse was 9.9 years, and the median number of cases of support for family caregivers of older adults who died of senility at home was 10.0.

**Table 1:** Demographic characteristics of participants.

		n or Mean±SD	% or (range)
		or Median	
Gender(n = 461)	Female	428	92.8
	Male	33	7.2
Age(n = 461)		49.0±9.0	(25.0‐85.0)
	<30	5	1.1
	30‐39	65	14.1
	40‐49	159	34.5
	50‐59	184	39.9
	60‐69	39	8.5
	70‐79	8	1.7
	80<	1	0.2
Years of work experience as nurse		23.7±9.2	(2.0‐50.0)
(n = 455)	<10	30	6.5
	10‐19	106	23.0
	20‐29	182	39.5
	30‐39	118	25.6
	40<	19	4.1
Years of work experience as visiting nurse		9.9±6.9	(0.5‐28.0)
(n = 452)	<10	231	50.1
	10‐19	170	36.9
	20<	51	11.1
Number of cases of support		22.9±55.1	(1‐1000)
(n = 428)		10.0	
	<5	101	21.9
	5‐9	61	13.2
	10‐49	199	43.2
	50‐99	46	10.0
	100<	21	4.6

Missing data were excluded from each analysis.

### Characteristics of the cases of end-of-life care for senility at home

Table 2 shows the characteristics reported by the visiting nurses in their experience of cases of senility at home. The mean age at the time of death was 89.8 years; 69.0% of the older adults were female, and the modal certification for “Requiring long-term care” at the time of death was 5 (certification of requiring long-term care insurance runs from 1–5, with 5 being the highest degree of care). The mean age of caregivers was 64.2 years; 74.8% of caregivers were female, 60.3% were children of the older adult individuals, 17.6% were spouses, and 13.4% were the spouses of children. The mean care period was 3.6 years and the median was 2.5 years.

**Table 2:** Summary of the end-of-life care case of senility at home.

		n or	% or (range)
		Mean ± SD	or Median
**Older adults**			
Gender(n = 455)	Female	318	69.0
	Male	137	29.7
Age at the time of death		89.8±7.7	(70‐112)
(n = 451)	<80	20	4.3
	80‐89	107	23.2
	90‐99	255	55.3
	100<	69	15.0
Support need at the time	Requiring long-term care 1	5	1.1
of death	Requiring long-term care 2	16	3.5
(n = 447)	Requiring long-term care 3	49	10.6
	Requiring long-term care 4	97	21.0
	Requiring long-term care 5	280	60.7
**Caregiver**			
Gender(n = 457)	Female	345	74.8
	Male	102	22.1
Age(n = 432)		64.2±11.6	(30.0‐91.0)
	<40	6	1.3
	40‐49	9	2.0
	50‐59	76	16.5
	60‐69	150	32.5
	70‐79	121	26.2
	80‐89	58	12.6
	90<	12	2.6
Relationship(n = 459)	Child	278	60.3
	Spouse	81	17.6
	Child spouse	62	13.4
	Grandson/Granddaughter	6	1.3
	Nephew/Niece	4	0.9
	Brother/Sister	4	0.9
	Other	1	0.2
	Two-persons	23	5.0
**Care period**			
Care period(n = 433)		3.6±3.9	(0.1‐25years)
		2.5	
	<6month	51	11.1
	6month‐11month	41	8.9
	1‐4years	218	47.3
	5‐9years	74	16.1
	10‐19years	43	9.3
	20<	6	1.3

Missing data were excluded from each analysis.

### Item analysis

The results of the item analysis are shown in Table 3. There were no items corresponding to the exclusion criteria, and factor analysis was therefore examined for all 15 items.

**Table 3:** Item analyses of the "Family caregiver needs-assessment scale for end-of-life care for senility at home".

							n = 461	
Item		Pass	Item	Population	Inter-item	Good-poor	Item-total	
No.	Item	efficiency^a^	Difficulty^b^	distribution^c^	correlation^d^	analysis^e^	correlation^f^	
1	Did the caregiver understand and adapt to the fact that feeding and water intake decrease along the course of senility?	3.8±0.4	0.2	99.0	-	0.000	0.716	**
2	Did the caregiver understand and adapt to edema and skin disorders caused by low protein?	3.7±0.5	0.9	96.8	-	0.000	0.741	**
3	Was there mutual understanding and communication with the elderly person?	3.7±0.5	1.1	96.8	-	0.000	0.438	**
4	Did the caregiver understand and adapt to the fact that the activity of the elderly person declines and the person tends to gradually fall asleep?	3.7±0.5	0.2	97.0	-	0.000	0.726	**
5	Did the caregiver understand and adapt to the patient’s psychiatric symptoms such as delirium, depression, strong anxiety, and BPSD※?	3.5±0.6	0.6	93.3	-	0.000	0.645	**
6	Was the caregiver able to properly use medical devices/assistive products?	3.3±0.7	0.4	88.5	-	0.000	0.568	**
7	Did the caregiver administer and use the amount of medicine necessary at an appropriate time?	3.4±0.7	0.6	91.3	-	0.000	0.617	**
8	Did the caregiver understand and adopt methods to relieve physical distress?	3.7±0.5	0.2	98.0	-	0.000	0.734	**
9	Did the caregiver correctly understand the medical condition and explanation of treatment provided by the physician?	3.7±0.5	0.0	98.2	-	0.000	0.623	**
10	Did the caregiver understand emergency situations in which he or she should make contact as well as how to initiate such contact?	3.8±0.4	0.2	99.1	-	0.000	0.565	**
11	Did the elderly person and his or her family members have quality of life/will to live?	3.5±0.6	0.6	95.4	-	0.000	0.559	**
12	Was the degree of fatigue experienced due to mental/physical condition of the caregiver in conjunction with caregiving within a permissible range?	3.7±0.5	0.4	98.0	-	0.000	0.511	**
13	Did the caregiver understand and accept the reality that death cannot always be prevented when attending to elderly patients?	3.6±0.6	1.1	95.4	-	0.000	0.604	**
14	Did the desires of the elderly person match those of his or her family members regarding end-of-life care?	3.7±0.5	0.2	97.0	-	0.000	0.616	**
15	Did the caregiver understand the symptoms indicative of imminent death from senility and have a system to provide care for the moment of death?	3.7±0.5	1.1	97.0	-	0.000	0.702	**

**:p<0.001

※BPSD: behavioral and psychological symptoms of dementia

Exclusion criteria of the item analyses.

a: Average score is under 2.0 point, "1.Not important", "2.Not important to a certain extent".

b: Percentage of don't know and NA is over 5% of the sample.

c:The percentage of 'Important' and 'Important to a certain extent' is lower than 85% of the sample.

d: Correlation is over 0.7.

e: Difference of the average score between most high-scoring group and most low-scoring group is not significant difference(p≥0.05).

f:The correlation coefficient between the item and the total of all the items(but with exception of the item) is less than 0.3 correlation coefficient.

### Exploratory factor analysis

The exploratory factor analysis results are shown in Table 4. The eigenvalues and scree plot suggested a two or three factor model. Exploratory factor analysis with promax rotation was repeated until the factor load of all items exceeded 0.41. As a result, items 3, 9, and 10 were excluded because the factor loading did not exceed 0.41 in any of the analyses. Exclusion of items with a loading less than 0.41 resulted in a two-factor solution, and 12 items on two factors were extracted for a final version of the scale. Factor 1 included six items (items 4, 11, 13, 14, and 15) interpretable as “Needs for adaptation to senility bereavement,” or death is natural providence, but caregivers cannot give up hope until they accept it. Factor 2 included six items (items 1, 2, 5, 6, 7, and 8) interpretable as “Needs for essential skills in supporting a dignified death by senility.”

**Table 4:** Factor analyses of the "Family caregiver needs-assessment scale for end-of-life care for senility at home"(final version).

					n = 231
	Cronbach's alpha coefficient		Factor 1 0.847	Factor 2 0.878	Total scale 0.908
No.	Item/[Factor]		Needs for adaptation to senility bereavement	Needs for essential skills in supporting a dignified death by senility	
14	Did the desires of the elderly person match those of his or her family members regarding end-of-life care?		0.861	-0.118	0.611
13	Did the caregiver understand and accept the reality that death cannot always be prevented when attending to elderly patients?		0.807	-0.091	0.556
15	Did the caregiver understand the symptoms indicative of imminent death from senility and have a system to provide care for the moment of death?		0.795	0.022	0.659
12	Was the degree of fatigue experienced due to mental/physical condition of the caregiver in conjunction with caregiving within a permissible range?		0.498	0.031	0.270
4	Did the caregiver understand and adapt to the fact that the activity of the elderly person declines and the person tends to gradually fall asleep?		0.456	0.316	0.512
11	Did the elderly person and his or her family members have quality of life/will to live?		0.417	0.257	0.393
7	Did the caregiver administer and use the amount of medicine necessary at an appropriate time?		-0.198	0.941	0.659
6	Was the caregiver able to properly use medical devices/assistive products?		-0.138	0.845	0.567
2	Did the caregiver understand and adapt to edema and skin disorders caused by low protein?		0.229	0.644	0.677
8	Did the caregiver understand and adopt methods to relieve physical distress?	0.255	0.581	0.614
5	Did the caregiver understand and adapt to the patient’s psychiatric symptoms such as delirium, depression, strong anxiety, and BPSD※?		0.212	0.528	0.483
1	Did the caregiver understand and adapt to the fact that feeding and water intake decrease along the course of senility?		0.310	0.505	0.574
Cumulative contribution %			48.4	54.8	
Factor correlation coefficients(r)		Factor 1	1.00		
		Factor 2	0.67	1.00	

Principal factor analysis with promax rotation.

※BPSD: behavioral and psychological symptoms of dementia

The factor loadings were greater than 0.41 for each factor. The cumulative contribution of the two factors explained 54.8% of the variance. Moreover, the correlation coefficient between the two factors was 0.67 (Table 4).

### Internal consistency and validity of the final scale

The Cronbach’s alpha coefficients were 0.847 for factor 1, 0.878 for factor 2, and 0.908 for the total scale (Table 4). The results showed that the scale had sufficient internal consistency.

**Fig 1:**
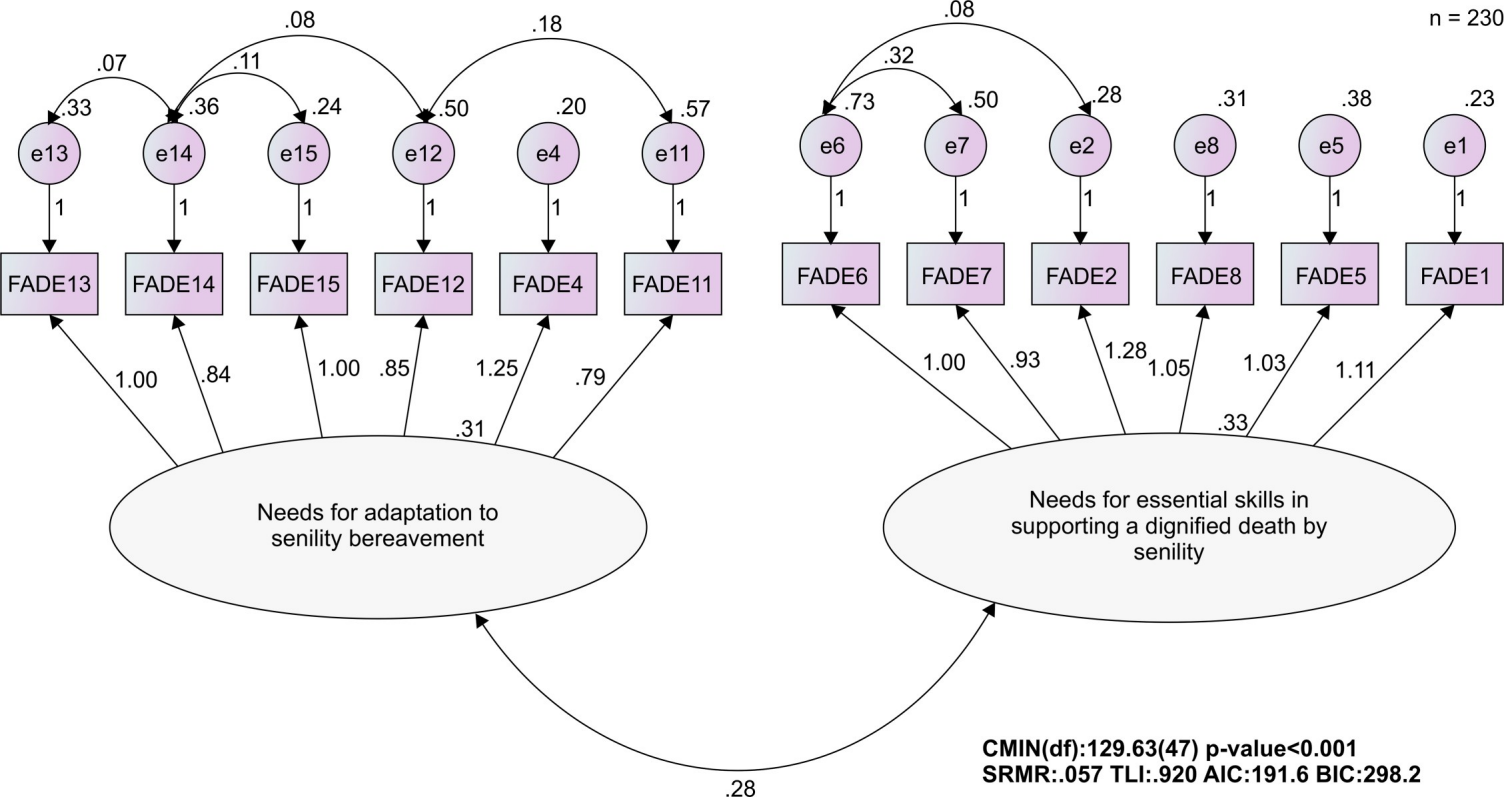
The confirmatory factor analysis of the FADE (final version).

These two factors were entered as latent factors in a confirmatory factor analysis model. In the initial model, SRMR = 0.080, TLI = 0.819, AIC = 312.8, and BIC = 398.7, which did not represent a good data–model fit. The model fit improved after modifying the model according to modification indices, adding error correlations for items 2 and 6, 6 and 7, 11 and 12, 12 and 14, 13 and 14, and 14 and 15 and incorporating the improved factors: SRMR = 0.057, TLI = 0.920, AIC = 191.6, and BIC = 298.2. These values were close to the criteria for an acceptable model, confirming the model’s the construct validity (Fig 1).

The Pearson’s correlation analysis showed correlations between the factors and STAS-J (items 4, 6, 7, and 9), with coefficients of 0.328–0.427 between Factor 1 and items 4, 6, 7, and 9; coefficients of 0.294–0.370 between Factor 2 and items 6 and 9; and coefficients of 0.259–0.427 between the total scale and items 4, 6, 7, and 9 (*p* < 0.001; Table 5).

**Table 5:** Construct validity of the "Family caregiver needs-assessment scale for end-of-life care for senility at home".

			n = 461
STAS―J	All	Factor 1 Needs for adaptation to senility bereavement	Factor 2 Needs for essential skills in supporting a dignified death by senility
item 4	.259**	.336**	.152**
item 6	.389**	.427**	.294**
item 7	.305**	.328**	.237**
item 9	.427**	.414**	.370**

**:p<0.001

Pearson's correlation coefficients between the Japanese version of the Support Team Assessment Schedule(STAS-J) item 4,6,7,9

item 4: Family anxiety

item 6: Family medical condition recognition

item 7: Communication between patients and their families

item 9: Medical staff communication to patients and families

## Discussion

The average age of the visiting nurses participating in this survey was 49 years, their average length of practice in nursing was 23.7 years, and their average length of practice in visiting nursing was 9.8 years. This is nearly identical to the profile of participants in the Survey on Home-Visit Nursing [29], who were an average of 47 years old, with 22.3 years of experience practicing nursing and 9.1 years of experience practicing visiting nursing. Thus, the sample was representative of the population of visiting nurses.

The originality of this research is that the development of the FADE scale focused on unrecognized needs for care provided by family caregivers of senescent older adult individuals, as well as its two foundational principles: adaptation to senility bereavement and preservation of the dignity of senescent older adult individuals. With regard to the reliability of the FADE scale, the Cronbach’s alpha coefficient for each factor and the scale as a whole indicated sufficient internal consistency. Correlations existed between the factors and the scale and the STAS-J, confirming the criteria-related relevance between the two measures. We assumed the correlations between Factor 2 and the STAS-J were rather small because the STAS-J targets families of patients with cancer. The results of a confirmatory factor analysis confirmed the validity of the concept behind the formulation of this scale. Thus, the FADE formulated through this research was judged to be a sufficient, reliable, and valid scale, capable of effectively assessing the sufficiency of needs of a family caregiver providing end-of-life care for a person with senility at home.

The first factor on the scale, “Needs for adaptation to senility bereavement,” comprised the needs presented by the senility process and the acceptance of death. Adaptation to such processes is always central to support. We assume that adapting to the process of senility may enable the caregiver to be calm at the time of death and provide appropriate end-of-life care sufficiently. Although aging and senility are laws of nature and death is unavoidable, caregivers often find it difficult to put aside their expectations and frequently experience significant emotional upheaval [30]. The six items that comprised this first factor, such as “acceptance of death,” “acceptance of senility,” and “development of a system for the moment of death,” highlighted the importance of slowly and gradually accepting and adapting to senility and death, even when experiencing anxiety at the passing of a loved one disturbs one’s emotional stability. With the support of visiting nurses to ensure that these needs are met, we expect caregivers may be able to attend to their duties in peace, and after the passing of their loved one, the fulfillment of these needs may even lessen their grief.

The second factor on the scale, “Needs for essential skills in supporting a dignified death by senility,” comprised the needs that enabled a caregiver to provide appropriate care. Appropriate care allows the older adult individual to meet his or her end painlessly [31]. For older adult individuals to have a painless end-of-life, proper medical care is necessary, and it is important for caregivers to acquire the required knowledge and skills to provide this care. Having a painless end-of-life shows respect to older individuals and allows a dignified death. The six items that comprised this second factor, such as “management and use of medical tools” and “treatment of symptoms,” highlighted the importance of assisting a caregiver in obtaining the knowledge, technology, and skills required to support older adult individuals as they live out their lives free of distress and pain. Fulfillment of these needs may help to ensure the older adult individual is respected and can pass away with dignity. The first FADE factor lists needs for caregiver adaptation while the second factor lists needs for care. These two factors are consistent with the two needs listed in previously published research (needs for the caregivers themselves and needs for carers’ support) and are suitable for use in end-of-life home-care practice [5].

The practical usability of this research in a clinical setting can be summarized by three points. First, by assessing the needs of a family caregiver of a person with senility, it enables the development of an individualized support approach. Visiting nurses can start to use the FADE when a prognosis of death due to senility in 3 to 6 months is given (decrease in feeding intake volume), and then use it once a month and when there is a change in condition. For each FADE item, the caregiver is interviewed by the visiting nurse about the situations of care, difficulty feeling, sense of burden, feelings regarding care and older adults, and relationships with other families and surroundings in daily practice. For example, for item 12 (“Is the degree of fatigue experienced due to the mental/physical condition of the caregiver in conjunction with caregiving within a permissible range?”), the following questions are included: “Are you getting enough sleep?”; “Do you have time to go to the hospital for yourself?”; and “Do you have time for yourself?” For item 15 (“Does the caregiver understand the symptoms indicative of imminent death from senility and have a system to provide care?”), the following questions are included: “Do you remember the doctor’s explanation that breathing changes when death is approaching?” and “Do you know whom to contact when breathing stops?” Furthermore, visiting nurses observe the daily practice of care and the condition of the older adults, comprehensively taking into consideration information obtained from other professionals involved in home support, and assess the sufficiency of needs. As a result, visiting nurses can reflect on the support for the needs in the care plan. Second, suggestions for projects and policies in the region in relation to what needs to be solved are obtained by constructing a system according to the needs clarified by individual support. Moreover, use of the FADE scale in conjunction with other facilities in the region can enable understanding of the strengths and weaknesses of the region from the view-point of satisfying the needs related to the death of senile patients in the region, and utilize it for system development. Third, this scale can be applied to both off-the-job and on-the-job training, and for personnel education on at-home-care for visiting nurses. Because visiting nurses mainly perform homecare alone and there is no standardized needs scale, it is left to the individual abilities of the visiting nurse to provide end-of-life care at home. By using the FADE, it is possible to unify the assessment contents from beginner to veteran. Visiting nurses can grasp the needs requiring assessment. In addition, through on-the-job and off-the-job training inside and outside the workplace on each need requiring clarification and on what kind of visiting nurse support is effective, it is possible to develop and share concrete support methods and give guidance to newly appointed visiting nurses. In turn, the education of visiting nurses is expected to improve the quality of at-home-nursing and thereby contribute to a qualitative improvement in homecare nursing.

The limitations of this research include the following. First, the response rate was low. Furthermore, the demographic characteristics of non-responders are unknown, so it is possible that the sample was biased. However, our response rate was relatively similar to that of a previously published study involving the same population [32]. In addition, 33% of facilities have reported no deaths at home over 1 year, which may explain the low response rate [33]. Moreover, because this study focused only on death from senility, the number of facilities may have been further limited. Second, the FADE was only sent to nurses and was evaluated through their retrospective recollection of a specific support case. This approach might have biased the results, imposed a burden on the respondent, and resulted in limited accuracy. Because the scale was not administered to any caregivers themselves in the present study, future research should administer the FADE directly to caregivers to obtain additional information. Third, this research was a cross-sectional study, and the predictive accuracy of the FADE scale is unknown. Consequently, future longitudinal research using the FADE should verify the prediction validity, for example, by clarifying the associations between assessment by the FADE scale and outcomes such as family satisfaction and dignified death from senility.

## Supporting information

S1 File Appendix. FADE English Version.
(PDF)

S2 File Appendix. FADE Japanese Version.
(PDF)

S3 File Appendix. Questionnaire (translated into English).
(PDF)

S4 File Appendix. Questionnaire (original).
(PDF)

## References

[1] Ministry of Health, Labour and Welfare, the White Paper on Annual Health, Labour and Welfare 2012 (2012) [cited 24 February 2019] https://www.mhlw.go.jp/wp/hakusyo/kousei/12/dl/1-06.pdf

[2] United Nations, Department of Economic and Social Affairs, World Population Prospects: The 2017 Revision [cited 11 March 2019] https://www.un.org/development/desa/publications/world-population-prospects-the-2017-revision.html.

[3] Petrini M, Cirulli F, D’Amore A, Masella R, Venerosi A, Carè A. Health issues and informal caregiving in Europe and Italy. Ann Ist Super Sanità. 2019:55(1): 41–50 10.4415/ANN_19_01_08 30968835

[4] Riedel M, Kraus M. Informal care provision in Europe: regulation and profile of providers. ENEPRI Research Report n. 96. 2011.

[5] Ewing G, Grande G. Development of a Carer Support Needs Assessment Tool (CSNAT) for end-of-life care practice at home: a qualitative study. Palliat Med. 2012;27(3): 244–256. 10.1177/0269216312440607 22450160

[6] Karlsson C, Berggren I. Dignified end-of-life care in the patients’ own homes. Nurs Ethics. 2011;18(3): 374–385. 10.1177/0969733011398100 21558113

[7] World Health Organization, ICD-11 for Mortality and Morbidity Statistics (2018) [cited 24 February 2019], https://icd.who.int/browse11/l-m/en

[8] Ministry of Health, Labour and Welfare, Demographics survey statistics (2018) [cited 31 July 2019] https://www.mhlw.go.jp/toukei/saikin/hw/jinkou/geppo/nengai18/index.html

[9] Ministry of Health, Labour and Welfare, Construction of Regional Comprehensive Care System (2015)[cited 24 February 2019] https://www.mhlw.go.jp/stf/shingi2/0000086278.html

[10] Ministry of Health, Labour and Welfare, Medical fee revision in (2016) [cited 28 January 2019] https://www.mhlw.go.jp/file/06-Seisakujouhou-12400000…/0000115980.pdf

[11] Ministry of Health, Labour and Welfare, Demographics survey statistics (2016) [cited 12 March 2019] https://www.mhlw.go.jp/toukei/saikin/hw/jinkou/kakutei17/index.html

[12] Ministry of Health, Labour and Welfare, Survey on end-of-life medical care (2010) [cited 24 February 2019] https://www.mhlw.go.jp/shingi/2008/10/dl/s1027-12e.pdf

[13] Visser G, Klinkenberg M, Broese V, Groenou M, Willems D, Knipscheer C, et al The end of life: informal care for dying older people and its relationship to place of death. Palliat Med.2004: 18(5). 468–77 10.1191/0269216304pm888oa 15332425

[14] Lynn J. Perspectives on care at the close of life. Serving patients who may die soon and their families: the role of hospice and other services. JAMA. 2001; 285(7): 925–32 10.1001/jama.285.7.925 11180736

[15] Swinkels JC, Broese van Groenou MI, de Boer A, Tilburg TGV. Male and female partner-caregivers’ burden: Does it get worse over time? The Gerontologist. 2018 10 12 10.1093/geront/gny132 [Epub ahead of print]. 30321338

[16] Murray S, Kendall M, Boyd K, Sheikh A. Illness trajectories and palliative care. BMJ. 2005; 330 (7498): 1007–11. 10.1136/bmj.330.7498.1007 15860828 PMC557152

[17] Aoun S, Grande G, Howting D, Deas K, Toye C, Stajduhar K, et al The impact of the carer support needs assessment tool (CSNAT) in community palliative care using a stepped wedge cluster trial. PLoS One. 2015;10(4): e0123012 10.1371/journal.pone.0123012 25849348 PMC4388632

[18] Aoun S, Deas K, Toye C, Ewing G, Grande G, Stajduhar K. Supporting family caregivers to identify their own needs in end-of-life care: Qualitative findings from a stepped wedge cluster trial. Palliat Med. 2015; 29(6): 508–17 10.1177/0269216314566061 25645667

[19] Perry L, Middleton S. An investigation of family carers’ needs following stroke survivors’ discharge from acute hospital care in Australia. Disabil Rehabil. 2011; 33(19–2):1890–1900 21314290 10.3109/09638288.2011.553702

[20] Matsumoto K, Takai K, Kirino M, Nakajima K. Measurement of care-related needs of family members caring for demented elderly patients at home. The Journal of Academy of Health Science. 2005; 8(3): 154–164

[21] Zarit S, Reever K, Bach-Peterson J. Relatives of the impaired elderly: Correlates of feelings of burden. Gerontologist. 1980; 20(6): 649–655 10.1093/geront/20.6.649 7203086

[22] Marwit S, Meuser T. Development of a short form inventory to assess grief in caregivers of dementia patients. Death Stud. 2005; 29(3): 191–205 10.1080/07481180590916335 15816111

[23] Weitzner M, Jacobsen P, Wagner H. The Caregiver Quality of Life Index-Cancer (CQOLC) scale: development and validation of an instrument to measure quality of life of the family caregiver of patients with cancer. Quality of life Research. 1999; 8(1–2): 55–63 10457738 10.1023/a:1026407010614

[24] Ito E, Tadaka E. Development of a Japanese Version of the Short-Form FAMCARE Scale for family caregivers of terminal cancer patients at home in Japan. Nippon Ronen Igakkai Zasshi. 2018; 55: 81–89. 10.3143/geriatrics.55.81 29503372

[25] Miyashita M, Matoba K, Sasahara T, Kizawa Y, Maruguchi M, Abe M et al Reliability and validity of the Japanese version of the Support Team Assessment Schedule (STAS-J). Palliat Support Care. 2004; 2(4): 379–385 16594400 10.1017/s1478951504040507

[26] Higginson I, McCarthy M. Validity of the support team assessment schedule: do staff’s ratings reflect those made by patients or their families?. Palliat Med. 1993; 7(3): 219–28 10.1177/026921639300700309 7505183

[27] Hospice foundation, STAS-J scoring manual third edition (2007) [cited 24 February 2019] https://www.hospat.org/stas-j.html

[28] Hu L, Bentler P. Cut off criteria for fit indices in covariance structure analysis: conventional criteria versus new alternatives. Structural Equation Modeling, 1999; 6: 1–55.

[29] Japanese Nursing Association, Visit Nursing Actual Survey Report of 2014 [cited 24 February 2019] https://www.nurse.or.jp/home/publication/pdf/2015/homonjittai-2014.pdf

[30] Stahl ST, Emanuel J, Albert SM, Dew MA, Schulz R, Robbins-Welty G, et al Design and rationale for a technology-based healthy lifestyle intervention in older adults grieving the loss of a spouse. Contemp Clin Trials Commun. 2017; 8: 99–105. 10.1016/j.conctc.2017.09.002 29170758 PMC5695565

[31] Albert R. End-of-life care: Managing common symptoms. Am Fam Physician. 2017 3 15;95 (6):356–361. 28318209

[32] Shimmura K, Tadaka E. Development of an interprofessional collaboration competency scale for children with medical complexity. BMJ Open. 2018;8(6): e019415 10.1136/bmjopen-2017-019415 29950458 PMC6020942

[33] Ministry of Health, Labour and Welfare. About remuneration, standard of visiting nursing (2017) [cited 15 March 2019] https://www.mhlw.go.jp/file/05-Shingikai-12601000…/0000184013.pdf.

