# Supplementary material for: Development of a family caregiver needs-assessment scale for end-of-life care for senility at home (FADE)
Source: PLoS One. 2019 Sep 11;14(9):e0222235. doi: 10.1371/journal.pone.0222235 (PMC6738926; doi:10.1371/journal.pone.0222235)
Supplement: S1 File — FADE English Version. (PDF) [file pone.0222235.s001.pdf]

S1 Appendix

FADE：Family Caregiver Needs-Assessment Scale For End-Of-Life Care For Senility At Home

0 – no unmet need, 1 – low unmet need, 2 – moderate unmet need, 3 – high unmet need

No	domain & item	evaluation			
**1. Needs for adaptation to senility bereavement**					
1	Does the desires of the older adult match those of his or her family members regarding end-of-life care?	0	1	2	3
2	Does the caregiver understand and accept the reality that death cannot always be prevented when attending to older adult?	0	1	2	3
3	Does the caregiver understand the symptoms indicative of imminent death from senility and have a system to provide care for the moment of death?	0	1	2	3
4	Is the degree of fatigue experienced due to mental/physical condition of the caregiver in conjunction with caregiving within a permissible range?	0	1	2	3
5	Does the caregiver understand and adapt to the fact that the activity of the older adult declines and the person tends to gradually fall asleep?	0	1	2	3
6	Does the older adult and his or her family members have quality of life/will to live?	0	1	2	3
**2. Needs for essential skills in supporting a dignified death by senility**					
7	Does the caregiver administer and use the amount of medicine necessary at an appropriate time?	0	1	2	3
8	Is the caregiver able to properly use medical devices/assistive products?	0	1	2	3
9	Does the caregiver understand and adapt to edema and skin disorders caused by low protein?	0	1	2	3
10	Does the caregiver understand and adopt methods to relieve physical distress?	0	1	2	3
11	Does the caregiver understand and adapt to the patient’s psychiatric symptoms such as delirium, depression, strong anxiety, and BPSD?	0	1	2	3
12	Does the caregiver understand and adapt to the fact that feeding and water intake decrease along the course of senility?	0	1	2	3
	No.1～12　total				

Saito M, Tadaka E, Arimoto A: Development of a family caregiver needs assessment scale for end-of-life care of senility at home (FADE), PLOS ONE. In review
