# Supplementary material for: Development of a family caregiver needs-assessment scale for end-of-life care for senility at home (FADE)
Source: PLoS One. 2019 Sep 11;14(9):e0222235. doi: 10.1371/journal.pone.0222235 (PMC6738926; doi:10.1371/journal.pone.0222235)
Supplement: S2 File — FADE Japanese Version. (PDF) [file pone.0222235.s002.pdf]

**S2 Appendix**
**FADE-J：Family Caregiver Needs-Assessment Scale For End-Of-Life Care For Senility At Home Japanese version**

# 日本語版家族介護者における老衰死の在宅看取りニーズ評価尺度

各項目について、家族介護者の認識、行動、状況ならびにその他得られた情報に基づき、総合的に評価してください。

| 領域 | No | 項目 | 0 充足している | 1 やや充足している | 2 やや充足していない | 3 充足していない |
|---|---|---|---|---|---|---|
| Ⅰ 老衰による死別への適応に向けたニーズ | 1 | 看取りに関する高齢者自身と家族の希望や意思決定が一致しているか | 0 | 1 | 2 | 3 |
| | 2 | 高齢者に死が避けられず来ることを認識し、向かい合うことができているか | 0 | 1 | 2 | 3 |
| | 3 | 老衰死に向けた臨死期の徴候を理解し、看取りに向けた体制が出来ているか | 0 | 1 | 2 | 3 |
| | 4 | 介護者の心身の健康状態や介護による疲労度は許容範囲か | 0 | 1 | 2 | 3 |
| | 5 | 活動性が低下し、徐々に眠っている時間が多くなることを理解し対処できているか | 0 | 1 | 2 | 3 |
| | 6 | 高齢者と家族での楽しみ・生きがいを持てているか | 0 | 1 | 2 | 3 |
| Ⅱ 尊厳ある老衰死を支えるためのニーズ | 7 | 適切なタイミングで必要量の頓用薬を使用し、管理できているか | 0 | 1 | 2 | 3 |
| | 8 | 医療機器・福祉機器を正しく使用し、管理できているか | 0 | 1 | 2 | 3 |
| | 9 | 低蛋白による浮腫やスキントラブルを理解し対処できているか | 0 | 1 | 2 | 3 |
| | 10 | 身体的苦痛の緩和方法を理解し対処できているか | 0 | 1 | 2 | 3 |
| | 11 | せん妄・うつ・強い不安・BPSD などの精神症状を理解し対処できているか | 0 | 1 | 2 | 3 |
| | 12 | 栄養・水分量が老衰の経過にそって減少することを理解し対処できているか | 0 | 1 | 2 | 3 |
| | | No.1～12　合計 | | | | 点 |

Saito M, Tadaka E, Arimoto A: Development of a family caregiver needs assessment scale for end-of-life care of senility at home (FADE), PLOS ONE. In review
