# Supplementary material for: Development of a family caregiver needs-assessment scale for end-of-life care for senility at home (FADE)
Source: PLoS One. 2019 Sep 11;14(9):e0222235. doi: 10.1371/journal.pone.0222235 (PMC6738926; doi:10.1371/journal.pone.0222235)
Supplement: S3 File — Questionnaire (translated into English). (PDF) [file pone.0222235.s003.pdf]

Questionnaire for
development of a family caregiver needs-assessment scale for end-of-life care of senility at home: FADE

1　We ask about you.

1）Gender

1　Male　　　　2　Female

2）Age

[　　　　　] years

3）Years of work experience

As nurse [　　　　　] years　　　　　　　As visiting nurse [　　　　　] years

4）The number of cases of support for family caregivers of older adults individuals who died of senility at home

[　　　　　]

2 Please answer the importance of each item of"Family caregiver needs assessment scale for end-of-life care of senility at home".The time to use this needs assessment scale is assumed to be the time when senility death is expected within about six months.

In addition, when importance can't be judged because the meaning of the item is not understood (it is difficult to understand), please put a✔ on ☐.

items	Important	Important to a certain extent	Not important to a certain extent	Not important	I don't no
1.Does caregiver understand and adapt to the fact that feeding and water intake decrease along the course of senility?	4	3	2	1	☐
2. Does caregiver understand and adapt to edema and skin disorders caused by low protein?	4	3	2	1	☐
3. Is there mutual understanding and communication between the older adult and caregiver?	4	3	2	1	☐
4. Does caregiver understand and adapt to the fact that the activity of the older adult declines and the person tends to gradually fall asleep?	4	3	2	1	☐
5. Does caregiver understand and adapt to the patient's psychiatric symptoms such as delirium, depression, strong anxiety, and Behavioral and Psychological Symptoms of Dementia (BPSD)?	4	3	2	1	☐
6. Is caregiver able to properly use medical devices/ assistive products?	4	3	2	1	☐
7. Does caregiver administer and use the amount of medicine necessary at an appropriate time?	4	3	2	1	☐
8. Does caregiver understand and adopt methods to relieve physical distress?	4	3	2	1	☐
9. Does caregiver correctly understand the medical condition and explanation of treatment provided by the physician?	4	3	2	1	☐
10. Does caregiver understand emergency situations in which he or she should make contact as well as how to initiate such contact?	4	3	2	1	☐
11. Do the older adult and his or her family members have quality of life/will to live?	4	3	2	1	☐
12. Is the degree of fatigue experienced due to mental/physical condition of the caregiver in conjunction with caregiving within a permissible range?	4	3	2	1	☐
13. Does caregiver understand and accept the reality that death cannot always be prevented when attending to older adult?	4	3	2	1	☐
14. Does the desires of the older adult match those of his or her family members regarding end-of-life care?	4	3	2	1	☐
15. Does caregiver understand the symptoms indicative of imminent death from senility and have a system to provide care for the moment of death?	4	3	2	1	☐

3 1) Please recall "one example of a case supporting the family caregiver of older adult who died senility at home" which you were in charge of in the past, and answer the following.

1.Gender and age at the time of death of older adult	1 Male 2 Female [ ]years
2.Support need at the time of death	Requiring long-term care 1 2 3 4 5
3.Gender and age of caregiver	1 Male 2 Female [ ]years
4.Relationship of caregiver	1 Spouse 2 Child 3 Child spouse 4 Grandson/Granddaughter 5 Brother/Sister 6 Nephew/Niece 7 Other ( )
5.Care period	[ ]years[ ]month

2) In the case of 1) above, please remember the family caregiver when the death of the older adult was supposed, and circle the numbers for the most appropriate needs satisfied

items	Satisfied	Satisfied to a certain extent	Unsatisfied to a certain extent	Unsatisfied
1.Does caregiver understand and adapt to the fact that feeding and water intake decrease along the course of senility?	0	1	2	3
2. Does caregiver understand and adapt to edema and skin disorders caused by low protein?	0	1	2	3
3. Is there mutual understanding and communication between the older adult and caregiver?	0	1	2	3
4. Does caregiver understand and adapt to the fact that the activity of the older adult declines and the person tends to gradually fall asleep?	0	1	2	3
5. Does caregiver understand and adapt to the patient's psychiatric symptoms such as delirium, depression, strong anxiety, and Behavioral and Psychological Symptoms of Dementia (BPSD)?	0	1	2	3
6. Is caregiver able to properly use medical devices/assistive products?	0	1	2	3
7. Does caregiver administer and use the amount of medicine necessary at an appropriate time?	0	1	2	3
8. Does caregiver understand and adopt methods to relieve physical distress?	0	1	2	3
9. Does caregiver correctly understand the medical condition and explanation of treatment provided by the physician?	0	1	2	3
10. Does caregiver understand emergency situations in which he or she should make contact as well as how to initiate such contact?	0	1	2	3
11. Do the older adult and his or her family members have quality of life/will to live?	0	1	2	3
12. Is the degree of fatigue experienced due to mental/physical condition of the caregiver in conjunction with caregiving within a permissible range?	0	1	2	3
13. Does caregiver understand and accept the reality that death cannot always be prevented when attending to older adult?	0	1	2	3
14. Does the desires of the older adult match those of his or her family members regarding end-of-life care?	0	1	2	3
15. Does caregiver understand the symptoms indicative of imminent death from senility and have a system to provide care for the moment of death?	0	1	2	3

3) In the case of 1) above, please remember the family caregiver when the death of the older adult was supposed, and circle the number of the situation that most fit for each item.

**1. FAMILY ANXIETY : Effect of anxiety on the family.**
0 None
1 Worry over changes. No physical or behavioural symptoms of anxiety.<br>Concentration not affected.
2 Waiting for changes or problems: on edge.<br>Occasional physical or behavioural symptoms of anxiety.
3 Anxious often. Physical/behavioural symptoms. Concentration markedly affected.
4 Completely and continuously preoccupied with anxiety and worries.<br>Unable to think of other matters.
**2. FAMILY INSIGHT : Family's awareness of the prognosis.**
0 Full awareness of prognosis.
1 Prognosis over or under estimated by up to 200%; e.g.<br>Thinking the prognosis is 6 months when it is likely to be 2 - 3.
2 Uncertain over patient becoming well or long prognosis; e.g.<br>'Some people with this die and he/she might too.'
3 Unrealistic; e.g.<br>Expecting to return to normal activity or work for a year when the time scale is only 3 months.
4 Expecting the patient to become completely well.
**3. COMMUNICATION BETWEEN PATIENT AND FAMILY**<br>**: Depth and openness of communication between patient and family.**
0 Communicating openly and honestly. Verbally and non verbally.
1 Communicating openly at some times or with some family members. OR<br>Sometimes with partner or with some members of family.
2 Acknowledge condition but discussion does not satisfy either the patient or family<br>who feels full implications are not discussed. OR Maybe with partner but not family.
3 Out of step, all discussions guarded.
4 Pretending.
5 Can not be evaluated due to cognitive decline or deep sedation
**4. COMMUNICATION PROFESSIONAL TO PATIENT AND FAMILY**<br>**: Depth of information given to patient and family, when they require this, from other professionals.**
0 Full information. Patient and family feel free to ask.
1 Information communicated but not clearly understood.
2 Facts given on request, patient or family would have liked more information.
3 Evasive, avoids true picture or some questions.
4 Avoids answering questions or visiting / gives incorrect information<br>which distresses patient and family.

This is the end of the survey. Thank you very much for your valuable time and cooperation.

Saito M, Tadaka E, Arimoto A: Development of a family caregiver needs assessment scale for end-of-life care of senility at home (FADE), PLOS ONE. In review
