# Supplementary material for: Development of a family caregiver needs-assessment scale for end-of-life care for senility at home (FADE)
Source: PLoS One. 2019 Sep 11;14(9):e0222235. doi: 10.1371/journal.pone.0222235 (PMC6738926; doi:10.1371/journal.pone.0222235)
Supplement: S4 File — Questionnaire (original). (PDF) [file pone.0222235.s004.pdf]

「家族介護者における老衰死の在宅看取りニーズ評価尺度の開発
(FADE：Family caregiver needs assessment scale for end-of-life care of senility at home)」
アンケート調査のお願い

平成30年10月1日（月）

までに郵便ポストにご投函くださいますようお願いいたします。

問1　あなたご自身のことについて伺います。[　] 内には数字をご記入下さい。

1）あなたの性別を教えて下さい。

1　男　　　　2　女

2）あなたの年齢を教えて下さい。

[　　　　] 歳

3）あなたの経験年数を教えて下さい。

看護師経験　[　　　　] 年　　　　　　訪問看護師経験　[　　　　] 年

4）あなたの「老衰死を迎えた高齢者の家族介護者の支援経験事例数」を教えて下さい。

おおむね　[　　　　] 例

問２　「家族介護者における老衰死の看取りニーズ評価尺度」の各項目の重要性について伺います。
あなたの考えに最も近いと思う数字１つに〇をつけて下さい。本ニーズ評価尺度を用いる時期は、概ね６ヵ月以内に老衰死が想定される時期とします。なお、項目の意味がわからない（わかりにくい）などにより重要性が判断できない場合には、□に✔印を入れて下さい。

【本尺度における用語の定義】
◆老衰死：加齢により生活全般において全介助であり他に致死的な病気の診断がついていない死亡
◆看取り：高齢者が個人として尊重され、尊厳を保ちながら最期まで穏やかに過ごせるようにすること
◆ニーズ：当事者が必要と感じている事柄や要望を踏まえ、訪問看護師が必要と判断している事柄

項目（案）	重要である	やや重要である	やや重要でない	重要でない	意味がわからない
1. 栄養・水分量が老衰の経過に沿って減少することを理解し対処できているか	4	3	2	1	□
2. 低蛋白による浮腫やスキントラブルを理解し対処できているか	4	3	2	1	□
3. 高齢者との間で意思疎通やコミュニケーションが図れているか	4	3	2	1	□
4. 活動性が低下し、徐々に眠っている時間が多くなることを理解し対処できているか	4	3	2	1	□
5. せん妄・うつ・強い不安・行動心理徴候（BPSD）などの精神症状を理解し対処できているか	4	3	2	1	□
6. 医療機器・福祉機器を正しく使用し、管理できているか	4	3	2	1	□
7. 適切なタイミングで必要量の頓用薬を使用し、管理できているか	4	3	2	1	□
8. 身体的苦痛の緩和方法を理解し対処できているか	4	3	2	1	□
9. 医師からの病状や治療に関する説明を正しく理解できているか	4	3	2	1	□
10. 連絡すべき緊急時の状況と連絡先を理解しているか	4	3	2	1	□
11. 高齢者と家族（主たる介護者およびそれ以外の家族を含む）での楽しみ・生きがいを持てているか	4	3	2	1	□
12. 介護者の心身の健康状態や介護による疲労度は許容範囲か	4	3	2	1	□
13. 高齢者に死が避けられず来ることを認識し、向かい合うことができているか	4	3	2	1	□
14. 看取りに関する高齢者自身と家族の希望や意思決定が一致しているか	4	3	2	1	□
15. 老衰死に向けた臨死期の徴候を理解し、看取りに向けた体制が出来ているか	4	3	2	1	□

問３　「家族介護者における老衰死の看取りニーズ評価尺度」の各項目の実践への適用性について伺います。

1）まず最初に、過去にご自身が担当された「在宅で老衰死を迎えた１事例」を想起し下記にご回答ください。

在宅で老衰死を迎えた１事例	
1. 高齢者の性別と<br>死亡時の年齢	1　男　　2　女　　[　　　　]歳・歳代
2. 死亡時の介護度	要支援 ・ 要介護１ ・ 要介護２ ・ 要介護３ ・ 要介護４ ・ 要介護５
3. 家族介護者の性別と年齢	1　男　　2　女　　[　　　　]歳・歳代
4. 家族介護者の続柄	1　配偶者　　2　子供　　3　子供の配偶者　　4　孫<br>5　兄弟または姉妹　　6　甥や姪　　7　その他（　　　　）
5. 介護期間	[　　　　]年[　　　　]ヶ月

2）上記、1）の１事例において、老衰死が想定された時点の家族介護者の状況を想起し、各項目のニーズがどの程度充足していたか（当てはまっていたか）について、もっとも当てはまる数字１つに○をつけて下さい。

項目（案）	充足している	やや充足している	やや充足していない	充足していない
1. 栄養・水分量が老衰の経過に沿って減少することを理解し対処できているか	0	1	2	3
2. 低蛋白による浮腫やスキントラブルを理解し対処できているか	0	1	2	3
3. 高齢者との間で意思疎通やコミュニケーションが図れているか	0	1	2	3
4. 活動性が低下し、徐々に眠っている時間が多くなることを理解し対処できているか	0	1	2	3
5. せん妄・うつ・強い不安・行動心理徴候（BPSD）などの精神症状を理解し対処できているか	0	1	2	3
6. 医療機器・福祉機器を正しく使用し、管理できているか	0	1	2	3
7. 適切なタイミングで必要量の頓用薬を使用し、管理できているか	0	1	2	3
8. 身体的苦痛の緩和方法を理解し対処できているか	0	1	2	3
9. 医師からの病状や治療に関する説明を正しく理解できているか	0	1	2	3
10. 連絡すべき緊急時の状況と連絡先を理解しているか	0	1	2	3
11. 高齢者と家族（主たる介護者およびそれ以外の家族を含む）での楽しみ・生きがいを持てているか	0	1	2	3
12. 介護者の心身の健康状態や介護による疲労度は許容範囲か	0	1	2	3
13. 高齢者に死が避けられず来ることを認識し、向かい合うことができているか	0	1	2	3
14. 看取りに関する高齢者自身と家族の希望や意思決定が一致しているか	0	1	2	3
15. 老衰死に向けた臨死期の徴候を理解し、看取りに向けた体制が出来ているか	0	1	2	3

3)　上記、1）の１事例において、老衰死が想定された時点の家族介護者を想起し、各項目がどのような状況であったかについて、もっとも当てはまる数字１つに○をつけて下さい。

**1. 家族の不安：不安が家族に及ぼす影響**	
0	不安なし
1	変化を気にしている。身体面や行動面に不安の徴候は見られない。集中力に影響はない。
2	今後の変化や問題に対して張り詰めた気持ちで過ごしている。時々、身体面や行動面に不安の徴候が見られる。
3	しばしば不安に襲われる。身体面や行動面にその徴候が見られる。物事への集中力に著しく支障をきたす。
4	持続的に不安や心配に強くとらわれている。他のことを考えることができない。
**2. 家族の病状認識：家族の予後に対する理解**	
0	予後について十分に理解している。
1	予後を２倍まで長く、または短く見積もっている。 例えば、2ー3ヶ月であろう予後を６ヶ月と考えている。
2	回復すること、または長生きすることに自信が持てない。例えば「この病気で死ぬ人もいるので、本人も近々そうなるかも知れない」と思っている。
3	非現実的に思っている。例えば、予後が３ヶ月しかない時に、１年後には普通の生活や仕事に復帰できると期待している。
4	高齢者が完全に回復することを期待している。
**3. 高齢者と家族とのコミュニケーション：高齢者と家族とのコミュニケーションの深さと率直さ**	
0	率直かつ誠実なコミュニケーションが、言語的・非言語的になされている。
1	時々、または家族の誰かと率直なコミュニケーションがなされている。
2	状況を認識してはいるが、その事について話し合いがなされていない。高齢者も家族も現状に満足していない。あるいは、パートナーとは話し合っても、他の家族とは話し合っていない。
3	状況認識が一致せずコミュニケーションがうまくいかないため、気を使いながら会話が行われている。
4	うわべだけのコミュニケーションがなされている。
5	認知機能の低下や深い鎮静により評価出来ない場合
**4. 高齢者・家族に対する医療スタッフのコミュニケーション：高齢者や家族が求めた時に医療スタッフが提供する情報の充実度**	
0	すべての情報が提供されている。高齢者や家族は気兼ねなく尋ねることができる。
1	情報は提供されているが、充分理解されてはいない。
2	要求に応じて事実は伝えられるが、高齢者や家族はそれより多くの情報を望んでいる可能性がある。
3	言い逃れをしたり、実際の状況や質問を避けたりする。
4	質問への回答を避けたり、訪問を断る。正確な情報が与えられず、高齢者や家族を悩ませる。

最後に、本研究に関するご感想ならびにご意見がございましたら、ご記入ください。

以上でアンケートは終了です。貴重なお時間を頂き、ご協力くださいまして、誠にありがとうございました。

Saito M, Tadaka E, Arimoto A: Development of a family caregiver needs assessment scale for end-of-life care of senility at home (FADE), PLOS ONE. In review
